# Comparative evaluation of MPTP and rotenone as inducing agents for Parkinson's disease in adult zebrafish: Behavioural and histopathological insights

**DOI:** 10.1016/j.toxrep.2025.102084

**Published:** 2025-07-12

**Authors:** Chetan Ashok, Naveen Kumar Rajasekaran, Srikanth Jeyabalan, Gayathri Veeraraghavan, Subalakshmi Suresh, Ramya Sugumar, Sugin Lal Jabaris, Vetriselvan Subramaniyan, Ling Shing Wong

**Affiliations:** a Department of Pharmacology, Faculty of Pharmacy, Sri Ramachandra Institute of Higher Education and Research (DU), Chennai, Tamil Nadu 600 116, India; b Centre for Toxicology and Developmental Research (CEFTE), Sri Ramachandra Institute of Higher Education and Research (DU), Chennai, Tamil Nadu 600 116, India; c Department of Pharmacology, Sri Ramachandra Medical College, Sri Ramachandra Institute of Higher Education and Research (DU), Chennai, Tamil Nadu 600 116, India; d Department of Pharmacology, Siddha Central Research Institute, Central Council for Research in Siddha, Anna Hospital Campus, Arumbakkam, Chennai 600 106, India; e Department of Biomedical Sciences, Sir Jeffrey Cheah Sunway Medical School, Faculty of Medical and Life Sciences, Sunway University, Selangor Darul Ehsan 47500, Malaysia; f Faculty of Health and Life Sciences, INTI International University, Putra Nilai, Nilai, Negeri Sembilan 71800, Malaysia

**Keywords:** Zebrafish, Parkinson’s disease, MPTP, Rotenone, Behavioural analysis, Histopathology

## Abstract

Parkinson's disease (PD), a prevalent neurodegenerative disorder, is marked by dopaminergic neuron loss and motor impairments. This study aimed to establish and compare PD models in adult zebrafish using two neurotoxins, MPTP and rotenone, evaluating their impact on behaviour and histopathology. Zebrafish were exposed to MPTP via intraperitoneal injection at two different doses or to rotenone in water for 21 days. Behavioural assessments, including Novel Tank Diving Test, bradykinesia, and C-bend response, revealed progressive motor and anxiety-like impairments, with rotenone exhibiting stronger locomotor effects. Histopathological analyses confirmed dose-dependent neurodegeneration in brain regions, with MPTP showing localized damage and rotenone causing widespread but milder effects. While both neurotoxins induced PD-like phenotypes, rotenone produced more pronounced locomotor deficits, whereas MPTP triggered anxiety-like symptoms. In conclusion, our study demonstrates that MPTP induces significant locomotor dysfunction along with anxiety-like symptoms, while rotenone strongly impacts locomotion with mild anxiety effects. Both neurotoxins exhibited maximum effects at their highest doses and over a similar time frame (Day 14 to Day 22). These findings highlight the distinct neurotoxic mechanisms of MPTP and rotenone and their relevance in modelling PD pathogenesis. The zebrafish model provides a robust platform for studying neurodegenerative diseases and testing therapeutic interventions. Further studies are required to explore the molecular mechanisms underlying their neurotoxic effects and to validate these models for long-term and translational research.

## Highlights

• Side-by-side neurobehavioral and histopathological analysis of MPTP and rotenone in zebrafish PD model.
• MPTP induced anxiety-like behaviour, while rotenone caused locomotor deficits in zebrafish model.
• Chronic exposure to MPTP and rotenone showed dose-dependent neurodegeneration in zebrafish brain.
• Rotenone exposure produced more severe bradykinesia and C-bend impairments than MPTP.
• Optimized zebrafish protocols aid neurotoxicology studies and PD model selection for Parkinson's research.

## Graphical Abstract

**Figure:**
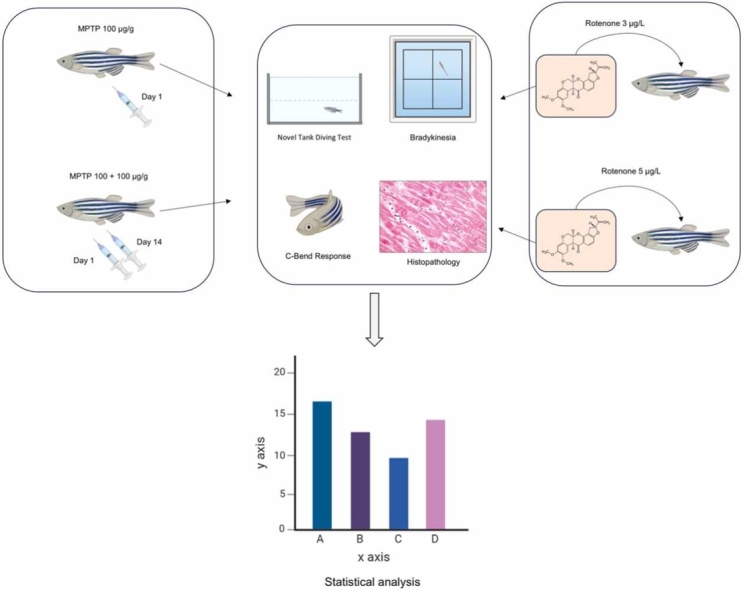

## 1 Introduction

Neurodegenerative disorders are becoming a significant public health issue, profoundly affecting the well-being and daily lives of patients and their caregivers alike. Parkinson’s disease (PD) is the second most common neurodegenerative disorder worldwide, following Alzheimer’s disease, and it primarily affects the elderly population [1]. This occurs as a result of the formation of Lewy bodies, which eventually leads to the loss of dopaminergic neurons in the substantia nigra pars compacta (SNpc) [2]. Located in the midbrain, the SNpc releases dopamine neurotransmitters to the striatum through the nigrostriatal pathway, a critical dopaminergic projection.

Research has consistently shown that the nigrostriatal pathway is the most extensively affected pathway in PD, sustaining significant damage. While the etiology of PD is predominantly sporadic, the loss of dopaminergic neurons in the substantia nigra is thought to be the result of a complex interplay among various factors, including mitochondrial dysfunction, oxidative stress, inflammation, and inadequate protein degradation [3], [4]. Despite significant advances in PD research, the underlying causes of nigral dopaminergic neuronal loss remain poorly understood. With a current global prevalence of approximately 9.4 million people, PD is expected to affect around 12 million individuals worldwide by 2040, according to projected estimates. PD is predominantly marked by motor impairments, including resting tremors, bradykinesia, and postural instability [1]. As the disease progresses into later stages, it can also give rise to non-motor symptoms, including cognitive impairments, sleep disturbances, gastrointestinal dysfunction, and depression [5].

The underlying causes of PD remain unclear, and its etiology is not yet fully understood. Genetic susceptibility and environmental exposures increase the risk of developing PD [6]. Current Parkinson's disease treatments are limited by the emergence of drug tolerance and unwanted side effects, highlighting the need for more effective therapeutic options. Studies have been vastly designed to find the pathogenesis and potential cure of this disease [7]. For decades, animal studies have played a crucial role in understanding PD. Researchers have employed animal models that mimic PD, using two primary methods to induce the disease: chemical induction through neurotoxins or genetic modification through transgenic approaches. Neurotoxins such as 6-hydroxydopamine (6-OHDA), 1-methyl-4-phenyl-1,2,3,6-tetrahydropyridine (MPTP), rotenone, and paraquat have been widely used in animal models [8], [9], [10].

Rotenone and MPTP are widely used neurotoxins for developing animal models of PD. Rotenone, a member of the rotenoid family, possesses a lipophilic structure that allows it to cross the blood–brain barrier with ease and accumulate in various organelles. It inhibits mitochondrial complex I and proteasomal activity, resulting in the generation of reactive oxygen species [11]. Rotenone has been found to promote alpha-synuclein aggregation, and recent research indicates that its toxicity is heightened by PARKIN and PINK1 gene knockouts, DJ-1 gene (PARK7) silencing, and alpha-synuclein overexpression [12]. Epidemiological research has shown that individuals with chronic exposure to rotenone have a higher incidence of Parkinson's disease compared to the general population [13].

MPTP is a type of meperidine analog formed as a by-product of the synthesis of 1-methyl-4-phenyl-4-propionoxypiperidine (MPP), synthetic heroin that is 5–10 times more potent than morphine [14]. MPTP easily crosses the blood–brain barrier, where astrocytes absorb it and quickly metabolize it into the toxic compound 1-methyl-4-phenylpyridinium iodide (MPP⁺) through the action of monoamine oxidase [2], [15], [16], [17]. MPP⁺ enters dopaminergic neurons through the dopamine transporter and is then partially stored in synaptic vesicles by the vesicular monoamine transporter. This process induces Parkinson-like behavioural, molecular, and proteomic characteristics in various animal models, including zebrafish [18]. In dopaminergic neurons, MPP⁺ accumulates within mitochondria, synaptic vesicles, and the cytoplasm, with a portion inhibiting mitochondrial complex I. While their exact mechanisms of action remain unclear, both MPTP and rotenone are known to effectively induce Parkinson’s disease-like symptoms [17], [19].

Rotenone and MPTP both suppress mitochondrial complex I activity, but they differ in their mechanisms of neuronal entry and their specific intracellular targets [20]. The majority of PD research using neurotoxin-induced models focuses on developing therapies to alleviate PD symptoms. To prevent confounding variables from influencing outcomes, it is crucial to establish and stabilize a robust PD model before initiating therapeutic interventions, thereby ensuring reliable and accurate results. In this study, zebrafish PD models induced by rotenone and MPTP were generated using 21-day exposure protocols and then compared in terms of neurobehavioral and neuropathological characteristics. While the administration routes differ for both the neurotoxins, they were selected to ensure optimal biological activity based established practices in zebrafish PD modelling and the known pharmacological properties of each compound. MPTP requires systemic delivery to reach the CNS effectively, while rotenone’s environmental relevance justifies waterborne exposure. Thus, although administration methods differ, our study compare the resulting neurotoxic phenotypes after equivalent chronic exposure periods.

The objective of this study was to establish a foundation for selecting and optimizing the most suitable zebrafish model for PD. Additionally, it aimed to develop a structured protocol using MPTP and rotenone to investigate PD pathogenesis and mechanisms while contributing to the advancement of potential treatment strategies. To the best of our knowledge, and based on a comprehensive review of existing literature, this study is the first to systematically compare two PD models across different doses in adult zebrafish. By integrating detailed behavioural assessments to evaluate functional outcomes alongside histopathological examinations to investigate tissue-specific pathological changes, this study provides novel insights into the dose-dependent effects of PD models in this vertebrate system. The findings from this research could enhance the understanding and refinement of PD animal model establishment in the future.

## 2 Materials and methods

### 2.1 Instrumentation

A 31 G needle attached to a 1.0 ml insulin syringe (U-100 Insulin 0.25 mm (31 G) x 6 mm Needle, Becton, Dickinson and Company, New Jersey, USA) was used for the intraperitoneal administration of MPTP in zebrafish. A Redmi Note 11 Pro+ Plus 5 G camera (Beijing, China) was utilized to capture the swimming behaviour, set to record at 720p resolution and 30fps frame rate. Video analysis was carried out using ANY-maze tracking software (version 7.45, Stoelting Co., India). Statistical analysis was performed using GraphPad Prism (version 9.5.1, GraphPad Software, CA, USA) on Windows.

### 2.2 Reagents and materials

The zebrafish were housed and maintained in tanks filled with filtered facility water throughout the experimental period. Their daily diet consisted of Optimum commercial fish food (Perfect Companion Group Co., Ltd, Samut Prakam, Thailand). The powdered MPTP was purchased from TCI Chemicals, India (Product number: M2690, purity >98 %, 209.72 g.mol^−1^) and Rotenone was purchased from Combi-Blocks, CA, USA (Cat# QD-3435, purity > 98 %, 394.41 g.mol^−1^). Dimethyl sulfoxide (DMSO) was purchased from SRL Chemicals (Cat# 43404, Mumbai, India). Normal saline (Otsuka Pharmaceutical Indian Pvt Ltd., Ahmedabad, India) was used to dissolve MPTP. Clove oil (Indian Pharmaceuticals, Old Washermanpet, India) was used for general anaesthesia. Methylene blue trihydrate (SRL Chemicals, Mumbai, India) was utilized as an antibacterial agent to support wound healing following injection.

### 2.3 Zebrafish care and maintenance

This study utilized fifty wild-type zebrafish (*Danio rerio*) of both sexes, aged 3–4 months, with an average weight of 450 ± 60 mg. The zebrafish were sourced from L.K. Aquarium, Chennai, India, and acclimatized for 14 days in a 60 L aquarium [61 cm (L) x 30 cm (W) x 39 cm (H)] at the Central Zebrafish Facility, Centre for Toxicology and Developmental Research (CEFTE), SRIHER, prior to experimentation. Both housing and experimental aquariums were maintained with filtered facility water and strictly controlled environmental conditions: temperature (26 ± 2°C), pH (7.1–7.4), hardness (60–75 g/L), light intensity (300 Lux), oxygen levels (8.0 mg/L), and relative humidity (60–80 %). Additionally, air bubbles provided aeration, and biofilter stones facilitated nitrate degradation throughout the study [21].

Illumination was provided by white fluorescence lamps, following a 14:10 light-dark cycle (lights on at 06:00), adhering to standard zebrafish care guidelines. Zebrafish were fed commercial adult food twice daily (09:00 and 16:00), with water exchanges every two days before feeding. This feeding regimen commenced two weeks prior to the main test, during acclimatization. The study's experimental paradigm was approved by the Institutional Animal Ethics Committee (IAEC) of Sri Ramachandra Institute of Higher Education and Research (Ref. no.: IAEC/72/SRIHER/889/2024) and conducted in compliance with Committee for Control and Supervision of Experiments on Animals (CCSEA) and Animal Research: Reporting of In Vivo Experiments (ARRIVE). The well-being of the fish was prioritized through appropriate nourishment and attentive husbandry practices.

### 2.4 Experimental setup

To ensure consistency across all the treatment groups, both male and female zebrafish were randomly distributed across five groups (Control, MPTP-A, MPTP-B, Rotenone-A, and Rotenone-B), minimizing any potential gender bias, each consisting of 10 fish (n = 10). Prior to experimentation, zebrafish were fed one hour in advance. The control group received 20 μL of 0.1 % DMSO solution through static immersion method for 21 days. In contrast, the MPTP-A group received a single injection of 100 μg/g MPTP on Day 1, whereas the MPTP-B group received cumulative doses of 100 + 100 μg/g MPTP, administered on Days 1 and 14. Rotenone-A and Rotenone-B groups were exposed to rotenone at 3 μg/L and 5 μg/L respectively in static method for 21 days (Table 1). Swimming behaviour tests were performed on Day 0, 14 and 22 after administration/exposure of the respective induction treatments. Throughout the 21-day experiment, the tank water was changed daily, and zebrafish were continuously exposed to rotenone or vehicle. Environmental conditions including temperature, pH, and light cycles were strictly controlled. Additionally, behavioural assessments and histopathological scoring were performed by investigators blinded to group assignments. Following behavioural recordings, zebrafish were humanely euthanized using a two-step procedure, i) anaesthesia with clove oil (1000 µl/L) until loss of motor coordination, and ii) immediate hypothermic shock (2–4°C) to minimize physiological stress. Death was confirmed by the cessation of opercular movements.

**Table 1:** Experimental design.

**Group name**	**Treatment**	**Details of Treatment**	**Number of animals**
Control	Vehicle (0.1 % DMSO)	Exposed continuously from Day 1 to Day 21	10
MPTP-A	MPTP (100 μg/g)	Single dose on Day 1	10
MPTP-B	MPTP (100 μg/g + 100 μg/g)	Dose on Day 1 (100 μg/g) and an additional dose on Day 14 (100 μg/g)	10
Rotenone-A	Rotenone (3 μg/L)	Exposed continuously from Day 1 to Day 21	10
Rotenone-B	Rotenone (5 μg/L)	Exposed continuously from Day 1 to Day 21	10
		**Total number of fish**	**50 Zebrafish**

**Fig. 1:**
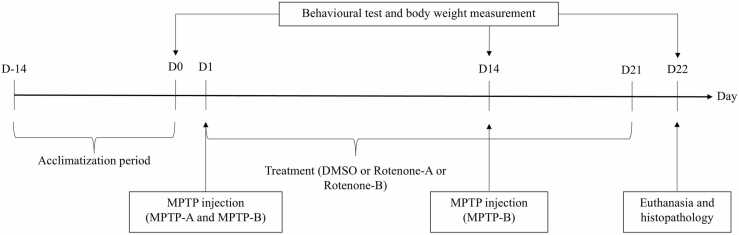
Experimental procedure timeline. MPTP injections were administered on Days 1 and 14. Rotenone exposure lasted for 21 days. Swimming behaviour tests were conducted on Days 0, 14, and 22.

Body weight (mg) measurements were collected from zebrafish on days 0, 14, and 22. During the 22-day study, the fish were maintained in a 4 L tank with dimensions 30.0 cm (L) x 15.0 cm (W) x 15.0 cm (H). Upon completion of the experiment, all zebrafish were sacrificed. The timeline summarizing the experimental design is depicted in Fig. 1**.**

### 2.5 MPTP induction

A study by Khairiah Razali et al. reported that the acceptable dose range of MPTP for adult zebrafish is 20 μg/g to 225 μg/g, with doses beyond 292 μg/g being lethal [22]. The ability of fish to repair and regenerate neuronal cells was observable from day 10 post-injection [23]. However, uncertainties remain regarding the optimal dose, the necessity of multiple doses, and the timing of post-injection assessments. Therefore, two different dose regimens of MPTP were chosen in this study to evaluate their effectiveness aiming to produce chronic neurodegeneration rather than transient behavioural impairments. MPTP powder was dissolved in normal saline to create a 5 µg/µl stock solution. Administration of MPTP in adult zebrafish often involves either intraperitoneal (i/p) injection or targeted delivery into the brain via intracerebroventricular injection [24], [25]. In this study, intraperitoneal (i.p.) injection was selected as the method for MPTP administration due to its established efficacy, reliable recovery rate, and cost-effectiveness [26], [27]. Individual fish were weighed by gently transferring them to a 100 ml beaker containing approximately one-third of the system water. The weight range of the fish was between 320 mg and 834 mg. The MPTP dosage was determined based on existing literature. MPTP-A group received only a single i/p dose of MPTP (100 µg/g, bw) on day 1 [23], while MPTP-B received two i/p doses of MPTP (100 µg/g, bw) on day 1 followed by 2nd dose (100 µg/g, bw) on day 14 [23].

After determining the appropriate dose, each fish was individually placed in a beaker containing 0.03 % (v/v) clove oil for anaesthesia. Once the fish reached anaesthesia stage 3 [28], it was carefully transferred onto a pre-soaked surgical sponge with a slit, positioned belly-up. A single dose of 10 μl of the working solution, containing 100 μg of MPTP per gram of body weight (100 μg/g bw), was administered intraperitoneally into the zebrafish using a 31 G needle connected to an insulin syringe (Supplementary Material, Fig. S1). The injection site was in the abdominal cavity, positioned just behind the pelvic fins, at an angle of 45˚ to the base of the pelvic fin [26], [27]. The injection method was validated by assessing five key indicators: (1) mortality, (2) bleeding at the injection site, (3) abnormal behaviour post-injection, (4) leakage of the injection solution, and (5) the observed effects of MPTP as critical markers of injection success [27]. The absence of recorded mortality, bleeding, abnormal behaviour, or solution leakage, along with the significant MPTP effect observed in the injected group, confirmed the effectiveness of the injection technique. Immediately following the procedure, zebrafish were placed in a recovery tank treated with an antibacterial solution (Methylene blue, SRL Chemicals, Mumbai, India) to promote wound healing. All fish were monitored during recovery for any adverse effects or injuries before being returned to their respective experimental tanks.

### 2.6 Rotenone exposure

Numerous studies have explored the neurodegenerative effects of rotenone at varying concentrations in adult zebrafish; however, ambiguity persists regarding its mortality rates and effectiveness. For example, research conducted by Glaucia Dal Santo et al. revealed that exposure to rotenone at a concentration of 3 µg/L led to a significant decline in SOD and GSH levels in adult zebrafish. Additionally, the study reported an increase in superoxide anion production and NADPH oxidase activity within the brain [29]. Conversely, another study reported no significant changes in zebrafish motility at a dose of 2 µg/L [30]. The literature further reveals inconsistencies in mortality rates at higher doses. Some studies report high mortality at 5 µg/L [31], while others identify 5 µg/L as an effective dose with manageable toxicity [30]. Additionally, another study found 7 µg/L to be the optimal dose, causing notable behavioural changes such as a 34 % decrease in swimming speed, a 34 % reduction in angular velocity, and an 836 % increase in freezing behaviour compared to the control group [32].

Given these discrepancies, there is a clear need for an effective and optimized dose of rotenone to develop a robust PD model with reduced mortality and a better understanding of its mechanism of action. Based on insights from these prior studies, we selected two different doses of rotenone for this study to address these gaps and ensure effective model development. Rotenone (Combi-Blocks, CA, USA) was dissolved in 0.1 % DMSO (SRL Chemicals, Mumbai, India) to prepare a stock solution of 1 µg/µl. Rotenone being light sensitive, immediately after preparation it was stored in a cool and dark place to prevent degradation from light exposure. The preferred route of rotenone administration is water exposure. Concentration of rotenone [33], [34] administered and exposure duration was based on previous studies. Rotenone-A and Rotenone-B groups were exposed to 3 µg/L [29] and 5 µg/L [30], [35] rotenone respectively. This concentration was obtained by adding 12 μL and 20 μL of the stock solution to separate 4 L volumes of fish system water. The solution was thoroughly mixed to ensure uniform distribution before reintroducing the fish.

The water in all tanks was replaced every 24 h, typically between 09:00 AM and 10:00 AM. Throughout the 21-day dosing period, the fish remained continuously exposed to rotenone and the vehicle solution. Feed was restricted before and 1 h after the exposure to the neurotoxins. Zebrafish was observed for mortality, morbidity and behavioural changes after daily exposure.

### 2.7 Behavioural test

Behavioural analysis was conducted for each fish individually on day 1, 14 and 22 to access the progress of behavioural alteration due to MPTP and rotenone exposure in zebrafish. Behavioural assessments, including the Novel Tank Diving Test (NTDT), evaluation of bradykinesia, and analysis of the C-bend response, were performed. The tanks were positioned on a stable surface and filled with filtered water from the facility. Video recording of the fish was performed using Redmi Note 11 Pro+ Plus 5 G camera. All behavioural assessments were conducted 1 h post-treatment exposure, with protocols carefully designed to minimize stress and ensure reliable evaluation of the subjects during testing. The recorded videos were saved in.mp4 format. Behavioural recordings were conducted between 09:00 and 14:00 to minimize potential disturbances associated with hormone fluctuations. Behavioural assessments were performed by investigators blinded to group assignments.

### 2.8 Novel Tank Diving Test (NTDT)

Each fish was individually introduced into the novel tank. The NTDT consists of a 5 L tank (30 × 15 × 15 cm) filled with water up to 12 cm [29]. Video recording by the lateral view was started immediately after the test fish were transferred into the novel tank and the behaviour of each zebrafish was observed and recorded for six minutes using the NTDT apparatus [36] and water temperature maintained at 26 ± 2˚C. Removable labels containing group details and fish number were attached onto the tank during the video recording. The apparatus was virtually segmented into two equal horizontal zones: the bottom and top sections. Various behavioural parameters were analysed, including total distance travelled (m), number of crossings, entries into the top zone, time spent in the top zone (s), average speed (m/s), total freezing episodes, distance travelled in the top zone (m), latency to the first entry into the top zone (s), time spent in the bottom zone (s), distance travelled in the bottom zone (m), and latency to the first exit from the bottom zone (s).

### 2.9 Bradykinesia

PD is predominantly marked by motor impairments, including bradykinesia. Bradykinesia is associated with the impaired ability to adjust the body position. To study the bradykinesia, fish was individually introduced into the 10 L experimental tank (25 ×30 × 31 cm) filled up to 14 cm with water. The base of the experimental tank was virtually divided into 4 quadrants with solid lines. Each quadrant had a dimension of 12.5 × 15 × 14 cm. A two-minute habituation period was provided, after which the number of quadrant changes made by the fish over a three-minute observation period was recorded. This parameter corresponds to the restricted body movement as in PD. Parameters including total immobile episodes, latency to start of first immobile episode (s), total time immobile (s), total distance travelled (m), average speed (m/s), absolute turn angle (°) and number of line crossings were analysed.

### 2.10 C-Bend response

The C-bend response in adult zebrafish, characterized by the rapid curvature of the body into a “C” shape followed by propulsion to escape a stimulus, was assessed to evaluate muscular rigidity, a primary symptom of PD. Individual adult zebrafish was introduced into a 100 ml beaker placed on a polyethylene foam stage. To elicit the C-bend response, the stage was gently tapped with a glass rod, delivering a vibrational stimulus. The stimulus was repeated six times for each fish, ensuring adequate rest intervals between stimuli to prevent habituation. The number of C-bends exhibited in response to each stimulus was recorded. The C-bend frequency directly correlates with the degree of muscular rigidity and motor responsiveness.

### 2.11 Histopathological evaluation

Whole brains were carefully excised and fixed in 10 % buffered formalin for 24 h at 4°C. Following fixation, the samples were dehydrated using a graded ethanol series, cleared with xylene, and embedded in paraffin wax for sectioning. The paraffin-embedded tissue was then cut into horizontal sections of 3–4 μm thickness using a rotary microtome (Leica R125 RM). The sections were subsequently dewaxed with xylene and rehydrated through a graded ethanol series, following standard protocols [37]. Following preparation, the sections were stained with haematoxylin–eosin (H&E) and examined under a B-510LD4 OPTIKA microscope (OPTIKA, Lombardy, Italy) with x20 magnification. Images were captured using a mounted OPTIKA P6FL Pro camera connected to OPTIKA Proview PC imaging software (Ver. 3.7) via USB 3.0 cable. The regions were identified based on the zebrafish brain topological atlas [38]. Histopathological alterations, including neuronal cell morphology, pyknosis, vacuolization, and immune cells, were assessed on each slide using a semiquantitative scoring system: 3, severe histopathological alteration; 2, moderate histopathological alteration; 1, mild histopathological alteration; 0, no histopathological alterations [36]. The scoring was conducted by experienced pathologists who were blinded to the experimental conditions. The grades from all slides were averaged to determine the final grade for each individual test fish.

### 2.12 Statistical analysis

The data were presented as mean ± SEM (standard error of the mean). Statistical analyses were conducted using GraphPad 9.5.1 (GraphPad Software, San Diego, CA, USA). A two-way ANOVA followed by Tukey’s multiple comparisons test was employed for data analysis. Statistical significance was defined as a p-value of less than 0.05 (p < 0.05). Each parameter was assessed in at least three independent experiments, each performed in triplicate.

## 3 Results

### 3.1 Novel Tank Diving Test (NTDT)

#### 3.1.1 Effect of MPTP and rotenone on movement and locomotion

**Fig. 2:**
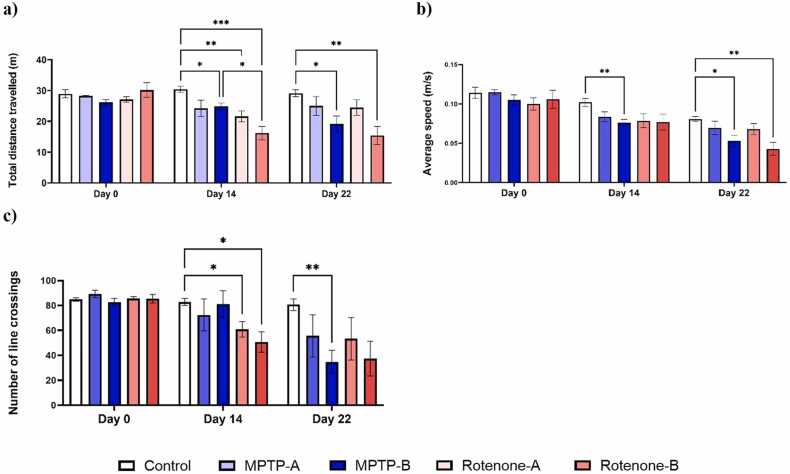
a) Total distance travelled in novel tank (m), b) Average speed (m/s), c) Number of crossings in novel tank. The data were expressed as mean ± SEM based on biological replicates (n = 10). Statistical significance was assessed using a two-way ANOVA followed by Tukey's post hoc test. Significance levels were denoted as *p < 0.05, **p < 0.01, and ***p < 0.001.

**Fig. 3:**
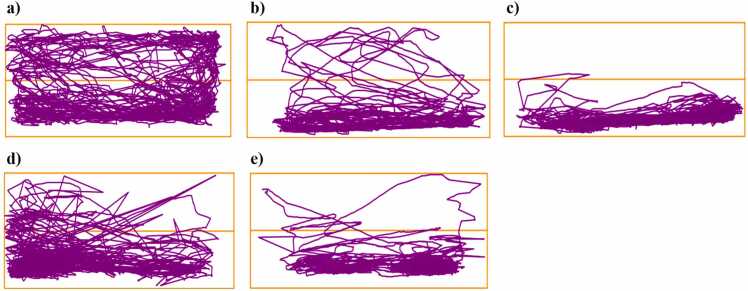
Locomotion trajectories of test zebrafish in novel tank after exposure to different treatments (n = 10). a) Control, b) MPTP-A, c) MPTP-B, d) Rotenone-A, and e) Rotenone-B.

Fig. 2. represents the effect of the MPTP and rotenone on the locomotor ability in zebrafish. Both MPTP and rotenone were able to significantly alter the distance travelled and the average speed in the NTDT apparatus by day 14. Notably a reduction of 11.46 % and 9.64 % was observed in the MPTP-A and Rotenone-A groups respectively on day 22, while a significant reduction of 26.73 % and 48.93 % in total distance travelled in the NTDT was observed in the MPTP-B and Rotenone-B groups respectively. Average speed was also reduced in the neurotoxin groups by 39.73 %, 49.58 %, 32.18 % and 59.02 % respectively for MPTP-A, MPTP-B, Rotenone-A and Rotenone-B groups. MPTP-A and Rotenone-A produced a similar reduction in the number of line crossings (37.74 % and 37.81 %), and similar results was observed at higher doses (57.93 % and 56.33 %). We also observed bradykinesia-like symptoms in rotenone-induced fish. This was evident by the significant reduction in total swimming distance. Tract plot of each group of zebrafish exploring NTDT are given in Fig. 3. Video clips depicting the overall behaviour of individual groups on day 22 are available as supplementary material (Supplementary Videos 1–5).

Supplementary material related to this article can be found online at doi:10.1016/j.toxrep.2025.102084.

The following is the Supplementary material related to this article Video S1, Video S2, Video S3, Video S4, Video S5

Video S1

Video S2

Video S3

Video S4

Video S5

#### 3.1.2 Effect of MPTP and rotenone on spatial preference

**Fig. 4:**
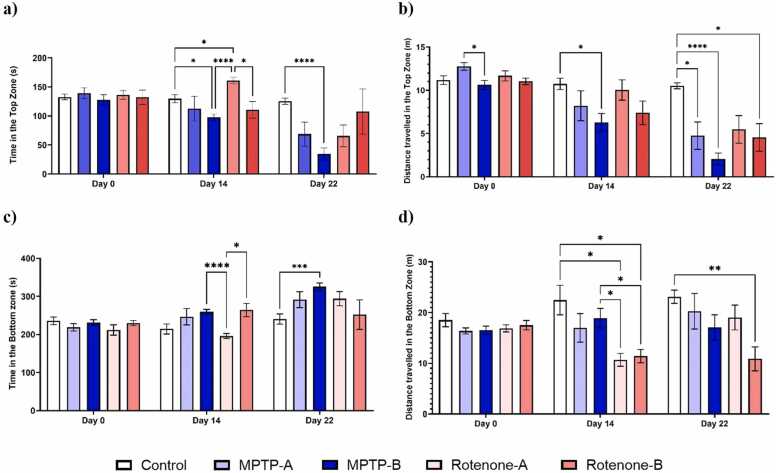
a) Time spent in top zone (s), b) Distance travelled in the top zone (m), c) Time spent in the bottom zone (s), d) Distance travelled in the bottom zone (m). The data were expressed as mean ± SEM based on biological replicates (n = 10). Statistical significance was assessed using a two-way ANOVA followed by Tukey's post hoc test. Significance levels were denoted as *p < 0.05, **p < 0.01, ***p < 0.001, ****p < 0.0001.

Fig. 4**.** shows the alterations in the spatial preference upon exposure to vehicle and different doses of MPTP and rotenone. By day 14, both neurotoxins induced fish showed an altered locomotor and spatial preference. MPTP-B showed a significant reduction in the time spent in the top zone (72.90 %). MPTP-A, Rotenone-A and Rotenone-B showed a reduction in the time spent in the top zone by 50.67 %, 51.73 % and 18.66 %. MPTP-A, MPTP-B and Rotenone-B observed a significant reduction in the distance travelled in the top zone by 62.80 %, 80.49 % and 58.80 % respectively. Interestingly, rotenone did not significantly impact zebrafish behaviour in the top and bottom zones. However, it did lead to a reduction in the distance travelled in the top zone compared to the control group. In contrast, the MPTP-B group exhibited a significant increase in time spent in the bottom zone, with a 40.45 % increase. While, Rotenone-B group observed a significant reduction in the distance travelled in the bottom zone (37.98 %). We also observed anxiety-like symptoms in MPTP-A and MPTP-B group. This was evident by the significant reduction in time spent and distance travelled in the top zone.

#### 3.1.3 Effect of MPTP and rotenone on anxiety-like behaviour

**Fig. 5:**
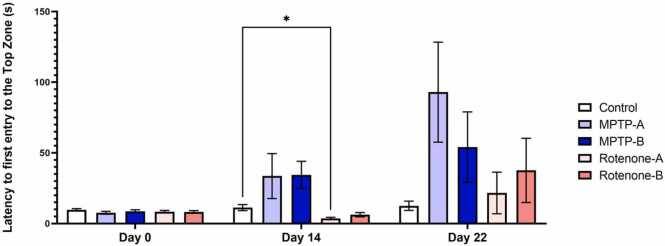
Latency to first entry to the top zone (s). The data were expressed as mean ± SEM based on biological replicates (n = 10). Statistical significance was assessed using a two-way ANOVA followed by Tukey's post hoc test. Significance levels were denoted as *p < 0.05.

Both MPTP and Rotenone exposure were able to delay the entry of zebrafish to the top zone inducing an anxiety-like behaviour. Day 0 observation show no variation in the entry of zebrafish to the top zone. On day14, MPTP exposure showed an increase in the avoidance towards the top zone and similarly MPTP-A and MPTP-B groups showed the highest avoidance to enter the top zone (12X and 7X times) as compared with the control and Rotenone exposed groups on day 22. Fig. 5**.** shows the latency (time delay) observed for the zebrafish to reach the top zone.

### 3.2 Bradykinesia

**Fig. 6:**
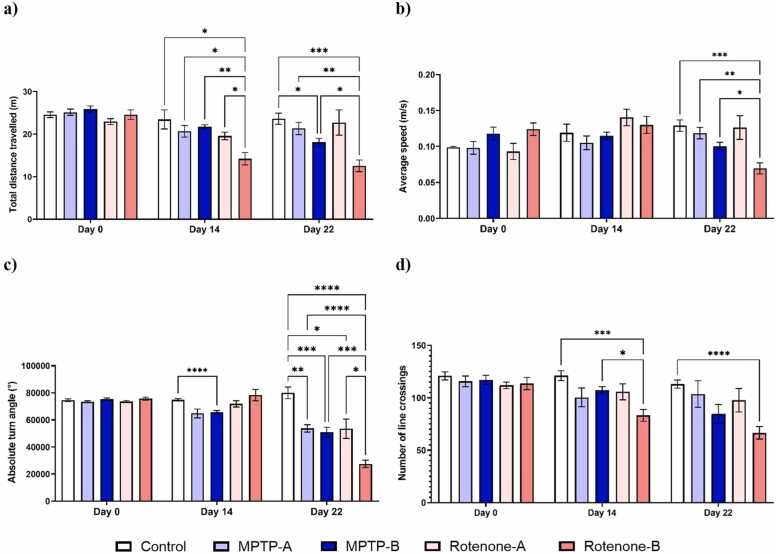
a) Total distance travelled in the bradykinesia tank, b) Average speed of zebrafish, c) Absolute turn angle (°), and d) Number of lines crossing between quadrants by zebrafish. The data were expressed as mean ± SEM based on biological replicates (n = 10). Statistical significance was assessed using a two-way ANOVA followed by Tukey's post hoc test. Significance levels were denoted as *p < 0.05, **p < 0.01, ***p < 0.001, ****p < 0.0001.

**Fig. 7:**
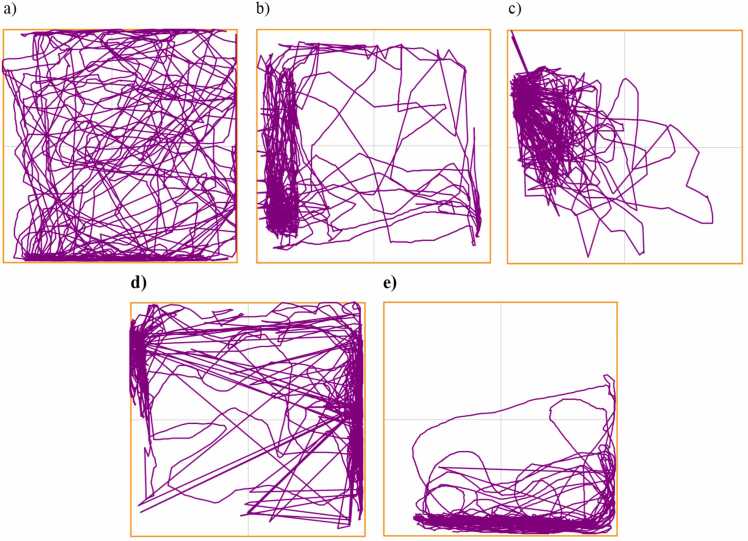
Locomotion trajectories of test zebrafish in bradykinesia tank after receiving various administrations. a) Control, b) MPTP-A, c) MPTP-B, d) Rotenone-A, and e) Rotenone-B.

Fig. 6 depicts the evaluation of bradykinesia-like behaviours by observing the absolute turn angle (˚), average speed (m/s), total distance travelled (m), and line crossings (n). Observable alterations in bradykinesia-like symptoms were seen by day 14. On day 22, MPTP-B (29.98 %) and Rotenone-B (48.94 %) groups observed a significant reduction in the total distance covered across the four quadrants. Rotenone-B group observed a significant decrease in the average speed with respect to control group, MPTP-A and MPTP-B, as evident for producing bradykinesia-like symptoms. Another factor to describe the swimming pattern of the zebrafish is the turning angle, it which is defined as the total degree of turning a zebrafish makes, regardless of the direction [39], [40]. Both MPTP and Rotenone exposure at both the concentration were able to significantly reduce the absolute turn angle (˚). Quantitatively, rotenone-treated groups, particularly Rotenone-B, demonstrated a more pronounced reduction in total distance travelled (48.94 %), average speed (43.83 %), and line crossings (41.47 %) compared to MPTP groups. Qualitatively, swimming patterns in the rotenone groups appeared more erratic and sluggish, consistent with impaired motor coordination, whereas MPTP-treated zebrafish exhibited moderate motor deficits but retained more consistent movement patterns. Fig. 7 represents the tract plot of individual zebrafish in the bradykinesia tank.

### 3.3 C bend response

**Fig. 8:**
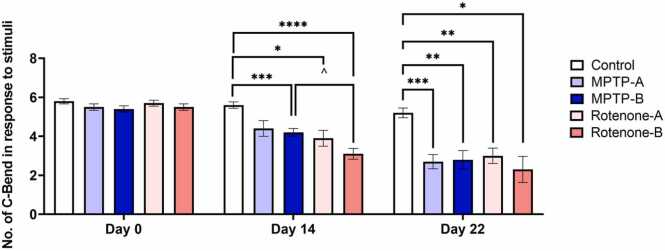
Number of C-Bend in response to external stimuli after exposure to different treatments. The data were expressed as mean ± SEM based on biological replicates (n = 10). Statistical significance was assessed using a two-way ANOVA followed by Tukey's post hoc test. Significance levels were denoted as *p < 0.05, **p < 0.01, ***p < 0.001, ****p < 0.0001.

Fig. 8 shows the C-Bend response of zebrafish upon external stimuli for different experimental groups. The response of zebrafish to external stimuli, including the presence and absence of C-bend reflexes, is shown in Supplementary Figure (Fig. S2). The control group showed consistent responses across all time points, with no significant changes in the number of C-bends. On Day 14, a significant decrease in the number of C-bends was observed in the MPTP-B, Rotenone-A, and Rotenone-B groups compared to the control group, with reductions of 48.15 %, 47.37 %, and 58.19 %, respectively. Among the treated groups, MPTP-B showed a more pronounced decline in C-bend frequency compared to MPTP-A (p < 0.05). Similarly, Rotenone-B exhibited a lower response compared to Rotenone-A (p < 0.05), suggesting a dose-dependent or cumulative effect of both MPTP and Rotenone exposure.

By Day 22, the reduction in C-bend responses became more pronounced across all groups. Significant reduction in C-bend response was observed in MPTP-A, MPTP-B, Rotenone-A and Rotenone-B groups, 51 %, 48 %, 47 % and 58 % respectively, as compared to the response on Day 0, indicating progressive motor impairment over time. Comparisons between MPTP-A and MPTP-B as well as Rotenone-A and Rotenone-B continued to show significant differences, reinforcing the trend observed on Day 14.

### 3.4 Changes in body weights after the administration of MPTP and Rotenone

**Fig. 9:**
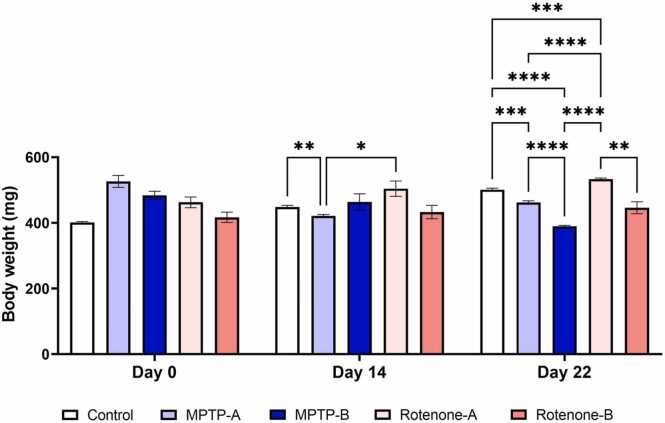
Body weight alteration in different experimental groups on day 0, day 14 and day 22. The data were expressed as mean ± SEM based on biological replicates (n = 10). Statistical significance was assessed using a two-way ANOVA followed by Tukey's post hoc test. Significance levels were denoted as *p < 0.05, **p < 0.01, ***p < 0.001, ****p < 0.0001.

In addition to assessing swimming behavior, we also evaluated differences in body weight at three time points: Day 0, Day 14, and Day 22. It is well established that experimental procedures, including injections, chemical exposures, and prolonged isolation, can induce stress in zebrafish, potentially leading to reduced appetite and subsequent weight loss. Since weight loss is often correlated with stress levels (Fig. 9), monitoring body weight provides valuable insight into the extent of stress experienced by the animals under the experimental conditions.

Both MPTP and rotenone resulted in a significant decrease in the body weights (p < 0.05) as compared to the control group. MPTP-A and MPTP-B caused a significant decrease in body weight (12.15 % and 19.54 %) in the zebrafish, proving its ability to increase stress level and loss of appetite. Rotenone at both concentrations didn’t seem to affect the body weigh as significant as the MPTP exposure caused but the weight gain was reduced to only 15.27 % and 6.99 % when compared to the body weight increase in the control group (24.87 %).

### 3.5 Histopathological alterations

**Fig. 10:**
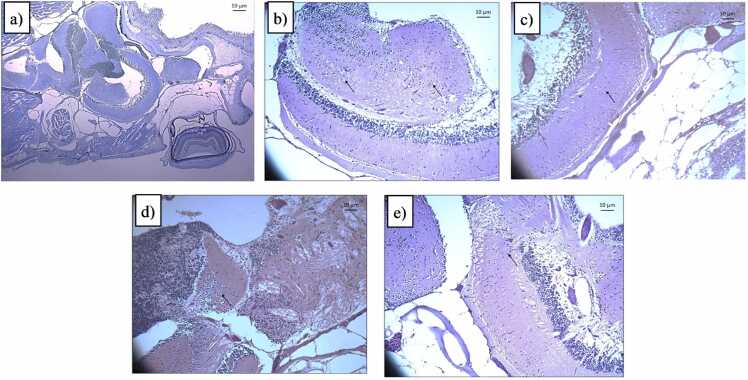
Histopathological alteration caused by MPTP and rotenone in zebrafish brain of different experimental groups a) Control, b) MPTP-A, c) MPTP-B, d) Rotenone-A, and e) Rotenone-B. All images captured at 20x magnification. Scale bar = 10 µm.

Histopathological analysis of brain tissues revealed neurodegenerative changes across experimental groups, Fig. 10**.** The control group showed no abnormalities, representing healthy baseline histology. In the MPTP-A group, mild neuronal degeneration, vacuolations, and congestion were observed in the corpus cerebelli, medulla, and olfactory bulb. Higher doses, MPTP-B exacerbated these effects, with moderate vacuolation and inflammation in cerebellar and medullary regions.

In the Rotenone-A group, minimal degeneration and mild vacuolation were noted in the olfactory bulb, corpus cerebelli, and medulla. At higher doses, Rotenone-B induced mild neuronal degeneration, vacuolation, and congestion across these regions. Both MPTP and rotenone showed dose-dependent neurodegenerative effects, with MPTP targeting cerebellar regions and rotenone causing milder, widespread changes. Higher rotenone dose demonstrated stronger neurotoxic impacts. These findings confirm the neurotoxic potential of MPTP and rotenone.

## 4 Discussion

The occurrence of PD is influenced by both genetic and environmental factors and is classified into two forms: familial and sporadic. Overall, ∼95 % of PD cases are sporadic and may be associated with environmental factors [41]. Currently, animal models of PD primarily include neurotoxin-based animal models and genetic animal models, each of which have advantages and disadvantages [17]. Although genetic animal models of PD provide precise insights into pathogenic mechanisms, they are often complex, time-consuming, and costly to develop. Compared with the genetic animal models, neurotoxin-based PD animal models can reproduce the behavioural changes and pathological characteristics of PD (mainly sporadic PD), and the modelling protocols are more straightforward [42]. The neurotoxin-based PD animal models reproduce the effects of toxic environmental factors associated with PD and have been widely used in model construction [43], [44], [45]. For example, rotenone is widely used as a crop insecticide and fish poison in fish culture systems [17], [46]. Studies have demonstrated that long-term, low-concentration exposure to toxicants may be an environmental factor that can lead to PD [47], [48], [49], [50]. It also has been reported that MPTP is associated with PD caused by drug abuse [51].

Therefore, this study focuses on the induction of neurotoxins in adult zebrafish to develop a reliable PD model, highlighting its practical significance for neurotoxin-based PD research. While prior studies have individually assessed MPTP or rotenone effects in zebrafish, our work is the first to systematically compare their dose-dependent effects side-by-side in adult zebrafish, something that is relatively underexplored in adult zebrafish, integrating behavioural endpoints with histopathological assessments over a chronic exposure period [31], [34], [52], [53], [54].

The study specifically assessed the effects of MPTP and rotenone administration on swimming behaviour and histopathological alterations in adult zebrafish. MPTP was administered intraperitoneally (i/p) in two dosing regimens: one group received a single dose of 100 µg/g on Day 1, while the other group received an additional second dose of 100 µg/g on Day 14 and similarly Rotenone was exposed to adult zebrafish in static method for 24 h daily over a period of 21 days. The findings of this study suggest that the selected dosage, administration route, and dosing interval of the neurotoxins effectively established a stable animal model while minimizing mortality. The survival rate of the MPTP groups was 100 % for both doses while Rotenone had a survival rate of 100 % at low dose (3 µg/L) and 90 % at high dose (5 µg/L), which provided feasible models to study PD. No significant gender-specific behavioural differences were observed, consistent with previous zebrafish neurotoxicity studies [55], [56], [57], [58], [59]

Their effects on swimming behaviour were assessed by Novel Tank Diving Test, Bradykinesia and C-Bend response (Day 0, Day 14 and Day 22). In this study, significant alterations in locomotor activity were observed, as evidenced by reduced swimming velocity, decreased distance travelled, and increased time spent in the bottom zone. These changes were detected on Day 14 and remained evident on Day 22. This suggests that, compared to the control group, zebrafish exposed to MPTP and rotenone exhibited slower swimming speeds and covered shorter distances.

MPTP administration reduced the average speed and total distance of adult zebrafish in NTDT. MPTP at higher dose (MPTP-B) showed a greater significant reduction (p < 0.05) in distance and mean speed as compared to the MPTP low dose (MPTP-A) and control group on Day 22. One of the most consistent findings in the adult zebrafish MPTP model is that locomotor alterations closely resemble those observed in human PD, reinforcing its relevance as a model for studying PD-related motor impairments. Decreases in both average velocity and average distance travelled during study periods were consistently observed [60].

MPTP at both doses (MPTP-A and MPTP-B) were able to alter the top zone behaviour in NTDT by significantly reducing the time spent in top zone and increasing the time spent in bottom zone and these findings were dose dependent. Since greater time spent in top zone and vice versa are related to anxiety-like symptoms in zebrafish [61]. MPTP, especially at higher dose (100 μg/g + 100 μg/g) developed anxiety more than locomotor dysfunction. This finding correlated with a previous study conducted with different doses of MPTP where zebrafish exposed to MTPT exhibited a reduction in the percentage of time spent by the fish on the top zone [2]. This is also in accordance with a few studies showing non-motor PD behaviours in adult zebrafish following induction with neurotoxins [16], [62]. Omar NA et al. [63] in another study found a substantial decrease in mean speed, total distance travelled, time spent in top zone and increase in latency to top zone in MPTP-injected zebrafish (100 μg/g of body weight) on the 3rd, 5th, and 10th days following injection. Our findings were in line with his findings. However, the study does not include data on additional swimming phenotypes, such as behavioural alterations in the bottom zone, latency to reach the top zone, and absolute turn angle. Additionally, it did not assess the number of crossings between the top and bottom zones in the Novel Tank Diving Test (NTDT) or between the four quadrants in bradykinesia assessment, which may provide a more comprehensive representation of swimming patterns compared to the time spent in a specific zone.

On the other hand, exposure to rotenone led to a reduction in locomotor activity, characterized by shorter swimming distances and lower mean velocity. Additionally, it altered the complexity of swimming patterns, as evidenced by its effects in the NTDT and bradykinesia assessments. Reduced locomotor activity, meaning a significant decrease in movement, is a key clinical manifestation of PD [64]. And Rotenone in a dose dependent manner caused a diminish locomotor activity resembling that of PD [65]. Our finding is in line with those of previous studies, which documented hypolocomotion in rotenone-induced animal models [33], [34], [66], [67]. Ünal et al. [68] in his study observed a significant decrease in the number of line crossings after exposure to rotenone for 4 weeks. Our findings were similar to the results of this study. However, observations on mean speed, absolute turn angle and distance travelled after rotenone exposure was missing.

Rotenone exposure at both doses was able to alter the top-bottom zone behaviour by reducing the time in the top zone and vice versa, but these effects were mild than MPTP exposure. This result was not consistent with increasing dose as Rotenone high dose (5 µg/L) group fish exhibited an improvement in time in top and bottom zones as compare to Rotenone low dose (3 µg/L) group. We assume this might be a result of the regenerative ability of zebrafish [69], [70] as Rotenone might not have caused neuron damage to a point at which zebrafish can’t recover. In contrast, MPTP exposure did not exhibit any signs of recovery in swimming behaviour at either dose. The locomotor impairments persisted on both Day 14 and Day 22 following MPTP administration, indicating a sustained effect of the neurotoxin on zebrafish movement. Surprisingly, distance travelled in both top and bottom zone were significantly reduced in MPTP and Rotenone groups, especially Rotenone exposure induced the maximum reduction in distance travelled, indicating an impairment in the locomotor activity when compared to control and MPTP exposures. PD is characterised by stiffness and reduction in ability to move causing reduced distance travelled in respective zones [71], [72]. Our findings correlate with a previous study conducted by Wang L et al. [73] where a similar significant reduction in total distance travelled and velocity was observed after exposure to neurotoxin, MPTP from 24 to 120 hpf.

Higher latency periods to enter the top zone has been related to anxiety-like behaviour and reduced exploratory activity [74], [75]. MPTP and Rotenone in a dose dependent manner affected the zebrafish’s normal entry into the top zone. MPTP at higher dose (100 μg/g + 100 μg/g) demonstrated a greater latency to enter the top zone on Day 22 as compared to the control and Rotenone exposure groups, results similar to that on Day 14. This indicates that higher doses of MPTP result in more pronounced neurotoxic effects, leading to increased anxiety-like behaviour in zebrafish. The consistent increase in latency observed across both time points suggests a progressive deterioration in dopaminergic neuronal function, reinforcing the dose-dependent nature of MPTP-induced neurotoxicity [76], [77].

PD is associated with numerous non-motor symptoms, i.e., mental illness, cognitive dysfunction, and pain [78], [79]. The anxiety symptoms in PD patients have also been well reported, with anxiety reported in 25.7 % of PD patients [80], [81]. The fact that the pattern of anxiety features in zebrafish and PD-specific features is similar may indicate the possibility that the neurochemical pathway plays a bigger role in inducing anxiety in PD patients than previously thought [82].

In bradykinesia apparatus, MPTP and Rotenone exhibited a significant reduction in the total distance travelled similar to results observed in the NTDT. Rotenone-B group observed the maximum reduction in the distance travelled as compared to MPTP-A, MPTP-B and control (p < 0.01, p < 0.05 and p < 0.001, respectively). Similar scenario can be observed in mean speed of zebrafish, where neurotoxins have affected the normal speed of zebrafish and Rotenone-B group exhibited the significant reduction in speed as compared to control, MPTP-A and MPTP-B. This indicated the ability of neurotoxins to alter the normal locomotion of zebrafish [83], [84].

The absolute turn angle, a key indicator of motor performance, was measured across different groups (Control, MPTP-A, MPTP-B, Rotenone-A, Rotenone-B) over three time points (Day 0, Day 14, and Day 22). The results clearly illustrate progressive motor impairment over time in the treated groups compared to controls, highlighting the neurodegenerative impact of both MPTP and rotenone on zebrafish locomotion. By Day 14, a significant increase in absolute turn angle is observed in the MPTP and rotenone-treated groups, suggesting an initial hyperactive response, potentially reflecting early compensatory mechanisms in the dopaminergic system [85]. However, by Day 22, a pronounced reduction in turn angle is evident, particularly in the Rotenone-B group, indicating severe motor dysfunction likely caused by dopaminergic neuron loss in the substantia nigra, as previously reported in zebrafish PD models [86].

The differential effects observed between the two concentrations of MPTP (MPTP-A and MPTP-B) and rotenone (Rotenone-A and Rotenone-B) are consistent with dose-dependent neurotoxicity. Higher doses resulted in greater motor impairment by Day 22, corroborating findings from previous studies demonstrating that both MPTP and rotenone exert their neurotoxic effects through mitochondrial dysfunction and oxidative stress, leading to apoptosis of dopaminergic neurons [87], [88]. Notably, rotenone exposure, especially at higher concentrations, produced more severe motor deficits than MPTP, which is consistent with its known mechanism of inducing complex I inhibition in mitochondria, leading to elevated reactive oxygen species (ROS) and subsequent neuronal death [89]. The significant differences between the control group and both MPTP and rotenone-treated groups (p < 0.05, p < 0.01, p < 0.001) indicate that zebrafish models are effective for replicating PD-like symptoms and can serve as a robust platform for studying the disease’s progression and potential therapeutic interventions. Behavioural endpoints, such as absolute turn angle, have been shown to be sensitive indicators of dopaminergic dysfunction in zebrafish, making them suitable for high-throughput drug screening [90].

The number of line crossings, a measure of locomotor activity, shows significant differences between control and MPTP/rotenone-treated zebrafish over time. On Day 14, a slight increase in locomotor activity is observed in MPTP-treated groups, which may reflect an initial hyperactivity or compensatory dopaminergic response to early neurodegeneration [85]. By Day 22, however, there is a marked reduction in line crossings, particularly in the high-concentration rotenone group (Rotenone-B), indicating severe motor impairment consistent with advanced dopaminergic neuron loss [86]. These findings align with previous studies showing that both MPTP and rotenone induce PD-like motor deficits by selectively targeting dopaminergic neurons. Rotenone’s stronger effect, as seen in the greater reduction in locomotion, may be due to its more potent inhibition of mitochondrial complex I and higher oxidative stress generation [91].

When comparing locomotor impairments, rotenone induced greater quantitative reductions in speed, distance, and angular movement than MPTP. This suggests a stronger dopaminergic suppression effect by rotenone [17]. In contrast, MPTP, while still impairing locomotion, showed more pronounced anxiety-like behaviors, indicating a differential neurotoxic profile. These distinctions reinforce the unique behavioral phenotypes associated with each neurotoxin and support the use of both models depending on the research focus, motor dysfunction or neuropsychiatric comorbidity. The statistically significant reduction in locomotor activity observed by Day 22 reinforces the validity of zebrafish as a model for studying PD progression. This further highlights its potential for evaluating therapeutic compounds aimed at mitigating PD-related motor impairments.

The C-bend response in zebrafish, a reflexive motor response to stimuli, is a reliable behavioural indicator of neural and motor system integrity. The Fig. 8 illustrates a significant decrease in the number of C-bends in zebrafish treated with MPTP and rotenone over time, with more pronounced deficits observed at Day 14 and Day 22 compared to the control group. This decline in reflexive behaviour is consistent with previous findings showing that neurotoxic compounds such as MPTP and rotenone induce dopaminergic neuron degeneration, which impairs motor function and reflexes [85]. On Day 14, both MPTP-treated and rotenone-treated groups show a moderate reduction in C-bend responses, indicating the early onset of neurodegeneration. By Day 22, the reduction is more pronounced, particularly in the high-concentration rotenone group (Rotenone-B), reflecting severe motor deficits associated with late-stage PD pathology [86]. The greater impact observed in rotenone-treated zebrafish is likely due to rotenone’s stronger inhibition of mitochondrial complex I, leading to elevated oxidative stress and apoptosis of dopaminergic neurons [92]. The significant dose-dependent reduction in C-bend responses across different neurotoxin groups highlights the progressive nature of neurodegeneration in this PD model.

The current procedure for C-bend response analysis aligns with studies highlighting the zebrafish acoustic startle response (ASR) as a model for assessing neuroplasticity and motor behaviours. Automated platforms such as Zebra_K analyze the kinematics of similar reflexive responses, noting the relevance of the C-bend as a short-latency response to external stimuli [93]. The manual approach employed in this study provides a straightforward and replicable method for measuring acute escape responses under controlled conditions. The observed C-bend frequency directly correlates with the degree of muscular rigidity and motor responsiveness. Similar to protocols used for startle response in zebrafish larvae and adults, the current setup ensures non-invasive, reproducible results while avoiding confounding factors such as stress habituation. These findings reinforce the utility of zebrafish as a model organism for studying PD progression and for evaluating the efficacy of potential neuroprotective agents targeting mitochondrial dysfunction and oxidative stress [94].

When evaluating the impact of neurotoxin exposure on animals, measuring the difference between initial and final body weights provides valuable insights. Body weight loss is a well-established indicator of stress levels, making it a relevant parameter for assessing the physiological effects of neurotoxin administration [95]. Therefore, monitoring body weight changes can serve as an indicator of the stress levels experienced by the animals under the experimental conditions. In this study, a significant reduction in body weights on Day 22 of the neurotoxin groups was observed as compared to control group fish body weight, indicating that both MPTP and Rotenone exposure has induced substantial stress levels that further reduced zebrafish appetites observed on Day 14 and 22. Nonetheless, further investigations are necessary to substantiate this claim, including the measurement of stress hormone levels and the expression of stress-related genes.

Histopathological analysis revealed dose-dependent neurodegenerative effects of MPTP and rotenone in zebrafish brains, aligning with their known neurotoxic mechanisms. Control zebrafish exhibited healthy histology, validating the experimental setup. MPTP caused progressive damage, with mild vacuolation and congestion in the MPTP-A group, escalating to moderate vacuolation and inflammation in the MPTP-B group. This corresponds to MPTP’s role in inducing oxidative stress and mitochondrial dysfunction through its toxic metabolite MPP⁺. Rotenone exposure also showed dose-dependent effects, with minimal degeneration at lower doses (Rotenone-A) and pronounced vacuolation and congestion at higher doses (Rotenone-B). Unlike MPTP’s targeted impact, rotenone caused milder but more widespread damage due to its broader cellular distribution.

The cerebellar and medullary regions were consistently affected, reflecting their vulnerability to oxidative and mitochondrial stress. These structural changes correlated with observed behavioural deficits like bradykinesia, validating these models for Parkinson’s disease research. Future studies should incorporate molecular analyses and extended exposure periods to further investigate the chronic progression and underlying mechanisms of neurodegeneration.

## 5 Conclusion

In summary, our findings demonstrate that systemic injection of MPTP (100 + 100 μg/g, bw) in adult zebrafish leads to a significant reduction in locomotor activity, alterations in swimming pattern, and abnormal swimming phenotypes. A key conclusion from this study is that MPTP has the potential to induce both locomotor dysfunction and anxiety-like symptoms, whereas rotenone primarily causes pronounced locomotor impairments with comparatively milder anxiety-like effects. Both MPTP and Rotenone reached their potential at their highest dose i.e., 100 + 100 µg/g for MPTP and 5 µg/L for Rotenone. Both neurotoxins produced discernible effects by Day 14 and continued till Day 22. The second key conclusion is that, beyond reducing locomotor activity, MPTP and rotenone administration induced motor incoordination in adult zebrafish. This was evident through alterations in swimming complexity and the presence of abnormal swimming phenotypes.

However, further research is necessary to completely unfold the underlying molecular mechanisms through which MPTP and Rotenone exerts its neurotoxic effects. Furthermore, an extended exposure study using MPTP and rotenone may be conducted to gain deeper insights into the underlying mechanisms and to allow histopathological changes to develop, making them comparable and relevant to clinical signs of Parkinson's disease. The use of appropriate animal models is crucial for researchers to investigate the underlying causes and mechanisms of diseases and to develop more effective treatments. This study demonstrated that the rotenone- and MPTP-induced PD zebrafish models exhibit distinct effects, highlighting important differences between them. Each model has its own advantages and limitations, and the choice between MPTP or rotenone, or a combination of both, should be guided by the specific objectives of the study. We believe this integrated comparison will aid in selecting appropriate models for specific research purposes. Overall, our study contributes to the development of novel strategies for development PD model by highlighting the role of MPTP and rotenone as potential neurotoxic agents.

## 6 Limitations

This study establishes a comparative framework using MPTP and rotenone to model Parkinson’s disease in adult zebrafish, offering valuable insights into behavioral and histopathological alterations. While the focus was on phenotypic characterization, future studies are planned to incorporate molecular validation techniques, such as tyrosine hydroxylase staining and dopaminergic neuron counts, to further substantiate neurodegeneration. The current design employed a single vehicle control, consistent with validated approaches in prior literature (e.g., Zhang et al., 2022); however, we recognize the value of adding route-specific vehicle controls in future protocols to strengthen interpretation. Additionally, although a 22-day timeline captured key neurotoxic effects, extending the observation period in follow-up studies will allow us to explore potential recovery patterns and neuroregenerative mechanisms in zebrafish. Together, these enhancements will build on the current findings and contribute to the development of refined and translationally relevant PD models.

## CRediT authorship contribution statement

**Chetan Ashok:** Writing – review & editing, Resources, Project administration, Investigation, Data curation, Conceptualization. **Naveen Kumar Rajasekaran:** Writing – review & editing, Methodology, Investigation, Formal analysis. **Srikanth Jeyabalan:** Writing – review & editing, Supervision, Methodology, Investigation, Conceptualization. **Gayathri Veeraraghavan:** Writing – review & editing, Supervision, Conceptualization. **Subalakshmi Suresh:** Writing – review & editing, Methodology, Investigation. **Ramya Sugumar:** Writing – review & editing, Methodology. **Sugin Lal Jabaris:** Writing – review & editing, Methodology. **Vetriselvan Subramaniyan:** Writing – review & editing, Methodology. **Ling Shing Wong:** Writing – review & editing, Methodology.

## Ethics approval and consent to participate

The present study was approved by the Institutional Animal Ethical Committee (IAEC) of Sri Ramachandra Institute of Higher Education and Research (DU), Chennai, India (Approval no. IAEC/72/SRIHER/889/2024; Chennai, India). This study was conducted in accordance with the CCSEA and ARRIVE guidelines to ensure rigorous and reproducible animal research. All experimental procedures involving zebrafish were designed and reported in compliance with CCSEA regulations and ARRIVE 2.0 recommendations.

## Funding

The project has been funded under GATE – Young Faculty Research Grant (14/PROVC/2024), Sri Ramachandra Medical College and Research Institute, Porur, Chennai for the year 2023–2024 and INTI International Research Fellowship Program (IIU/HR/JL/NHZ/12566/23), INTI University, Malaysia for the year 2023-2025.

## Declaration of Competing Interest

The authors declare that they have no known competing financial interests or personal relationships that could have appeared to influence the work reported in this paper.

## Data Availability

Data will be made available on request. The data that support the findings of this study are available on request from the corresponding author.
