# Supplementary material for: Comparative evaluation of MPTP and rotenone as inducing agents for Parkinson's disease in adult zebrafish: Behavioural and histopathological insights
Source: Toxicol Rep. 2025 Jul 12;15:102084. doi: 10.1016/j.toxrep.2025.102084 (PMC12302763; doi:10.1016/j.toxrep.2025.102084)
Supplement: Supplementary file 6 — Supplementary material [file mmc1.docx]

**SUPPLEMENTARY DATA**


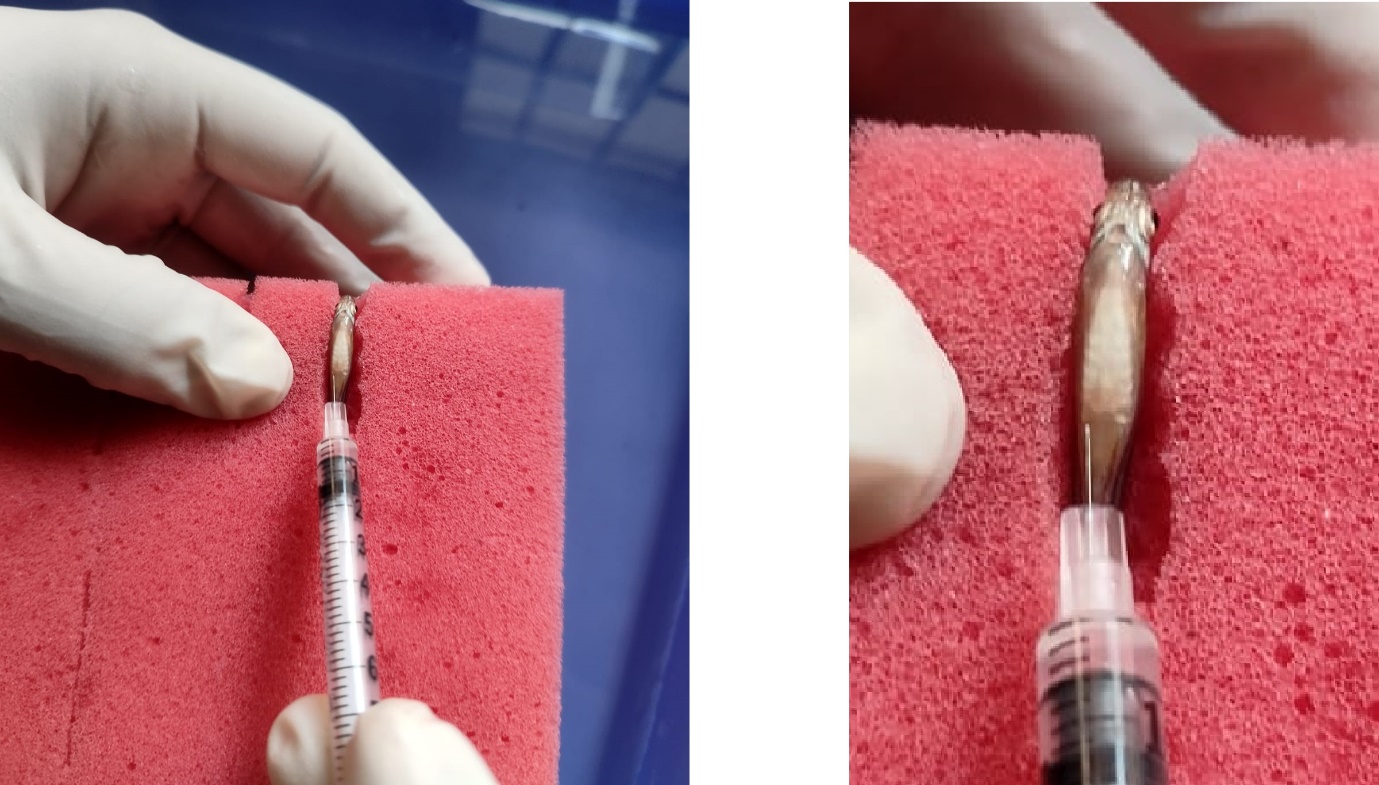
**Fig. S1.** A schematic representation of the experimental setup for intraperitoneal injection. The anesthetized zebrafish was positioned with its ventral side facing upward on a chilled sponge. The magnified section highlights the precise injection site. A 31G needle connected to an insulin syringe was used to administer MPTP to the MPTP-A and MPTP-B groups

| 1. **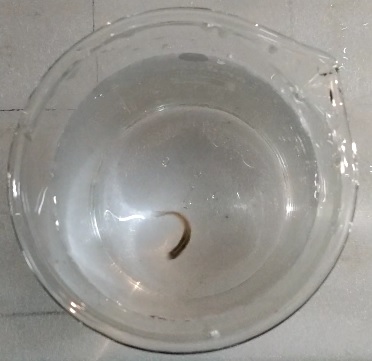** | 1. **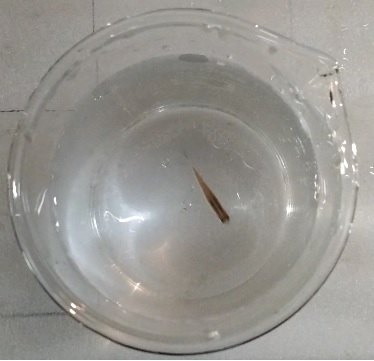** |
| --- | --- |

**Fig. S2.** a) C-Bend response of adult zebrafish upon external stimuli, b) No response upon external stimuli
